# Nitrogen-Doped Weathered Coal for the Efficient Adsorption of Lead: Adsorption Performance and Mechanisms

**DOI:** 10.3390/molecules29235589

**Published:** 2024-11-26

**Authors:** Xiaojing Chen, Xiaobing Jin, Chi Zhang, Zile Jiao, Zhiping Yang, Ke Wang, Jianhua Li, Qiang Zhang

**Affiliations:** 1 Institute of Eco-Environmental Industry Technology, College of Resources and Environment, Shanxi Agricultural University, Taiyuan 030031, China; jinxiaobing@sxau.edu.cn (X.J.); 20233485@stu.sxau.edu.cn (C.Z.); yzpsx0208@163.com (Z.Y.); wangke@sxau.edu.cn (K.W.); jianhua0119@163.com (J.L.); 2 Soil Health Laboratory in Shanxi Province, Taiyuan 030031, China; 3 Institute of Loess Plateau, Shanxi University, Taiyuan 030006, China; 4 College of Environmental Science and Engineering, Beijing Forestry University, Beijing 100083, China; jzl2573657313@163.com

**Keywords:** nitrogen-containing group, weathered coal, lead, adsorption property, complexation

## Abstract

The development of widely sourced and efficient adsorbents is crucial for the adsorption of lead from wastewater. A novel adsorbent, N-doped weathered coal (NWC), was prepared in this study using weathered coal as the precursor and triethylenetetramine (TETA) as the N-source. The adsorption performance and behavior of Pb(II) on NWC were investigated using batch adsorption experiments. The results demonstrated that NWC has an efficient adsorption performance towards Pb(II), with a maximum monolayer adsorption capacity of 216.32 mg g^−1^ (25 °C). The adsorption process was spontaneous and endothermic, and the importance of chemisorption was observed. The adsorption mechanisms of NWC were also analyzed based on its physicochemical structure before and after the Pb(II) adsorption and desorption experiments. The N and O functional groups, acting as electron donors, promoted coordination with Pb(II), making complexation the dominant mechanism. Its contribution to the adsorption mechanism could reach 44.81%. NWC is a promising material for both wastewater treatment and the resource utilization of weathered coal.

## 1. Introduction

Accelerated industrialization generates large amounts of wastewater, and the protection and rational utilization of water resources has become a global concern [1]. Lead is widely used in mining, smelting, battery manufacturing, chemical processes, and other industries, which inevitably causes the generation and discharge of lead-contaminated wastewater [2]. Pb(II) is a highly toxic and persistent environmental pollutant that strongly bioaccumulates. It can enter and accumulate in plants and in the human body via the food chain, bringing about irreversible harm to plant growth and human health [3,4]. Therefore, it is necessary to effectively treat Pb(II) in wastewater prior to direct discharge. Numerous techniques, such as coagulation, membrane filtration, reverse osmosis, and adsorption, have been developed for treating Pb(II)-contaminated wastewater [5,6]. Among these, adsorption is effective, easy to use, and may result in energy savings compared to alternative methods [7,8]. Widely sourced and efficient adsorbents are constantly being developed [9].

China produces approximately 10 billion tons of weathered coal (WC) [10]. Weathered coal denotes coal that is exposed on the surface or situated in the shallow layers of the surface. It is created through the weathering of anthracite, bituminous coal, and lignite, and it is a low-quality coal [11]. Because of its high oxygen content and low thermal energy, it has little value as a fuel and raw coking material, and only a part of it has been developed as a humic acid fertilizer [12,13]. A large amount of waste will be generated by weathered coal if it is not properly developed and utilized. The surface of weathered coal exhibits pores and includes active oxygen-bearing functional groups like carboxyl groups (−COOH) and hydroxyl groups (−OH), which can form complexes with metal cations and can be used to adsorb metal cations [14,15]. In particular, it is an ideal raw material for preparing heavy metal adsorbents. However, due to the low content of active functional groups, its adsorption capacity for heavy metals is limited. Therefore, increasing the quantity of active functional groups within weathered coal and improving its adsorption capacity for heavy metals are important for the effective application of weathered coal as an adsorbent for lead ions.

Nitrogen-containing groups have been reported to exhibit a good adsorption capacity for adsorbing metal ions [16]. Owing to its high electronegativity, nitrogen doping of the carbon framework can change the local electron density and polarity of the carbon material, thereby increasing its interaction with heavy metal ions [17,18]. However, it remains unclear how the introduced nitrogen-containing functional groups affect the adsorption performance of weathered coal for Pb (II).

In this study, N-doped weathered coal (NWC) is prepared using weathered coal as the precursor and triethylenetetramine (TETA) as the N source. Based on batch adsorption experiments, the adsorption performance and behavior of Pb(II) on NWC were systematically researched. The possible adsorption mechanism of Pb (II) by NWC is discussed using various characterization techniques and desorption experiments. This research aims to provide valuable insights into the potential application of weathered coal as an affordable and efficient adsorbent for Pb (II) extraction. The findings of this research may aid in the invention of efficient adsorbents for Pb(II)-contaminated wastewater and provide reference and theoretical support for the resource utilization of weathered coal. This research contributes to the body of knowledge on Pb(II) adsorption and also promotes non-renewable resource efficiency.

## 2. Results and Discussion

### 2.1. Physicochemical Structure of NWC

The physicochemical structure of NWC is closely related to its adsorption performance and was investigated using various characterization techniques.

#### 2.1.1. Physical Structure

**Figure 1:**
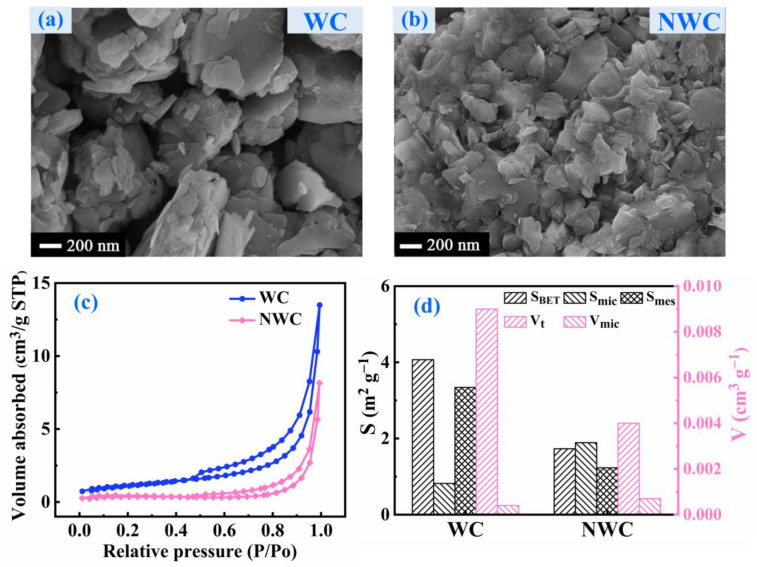
SEM images of WC (**a**) and NWC (**b**); N_2_ adsorption–desorption isotherms of WC and NWC (**c**); pore structure parameters of WC and NWC (**d**).

The surface morphology of NWC was examined with SEM, as shown in Figure 1b. After nitrogen doping, the surface of NWC became more fragmented. As a result, the particles became smaller and more uniformly distributed. The gaps between the particles were evenly covered, which may have resulted from the destruction of the WC structure owing to ultrasonic modification and the adhesion and occupation of multiple vinyl and amino groups of triethylenetetramine [19]. This resulted in a decrease in the N_2_ adsorption–desorption isotherms of NWC (Figure 1c). The specific surface area (S_BET_), mesopore surface area (S_mes_), and total pore volume (V_t_) of NWC were lower than those of WC (Figure 1d and Table S1). However, the micropore surface area (S_mic_) and micropore volume (V_mic_) of NWC increased by 130% and 75%, respectively. This may also be attributed to the destruction of the WC structure caused by ultrasonic modification and the adhesion and occupation of multiple vinyl and amino groups of triethylenetetramine [19].

#### 2.1.2. Surface Chemistry

**Figure 2:**
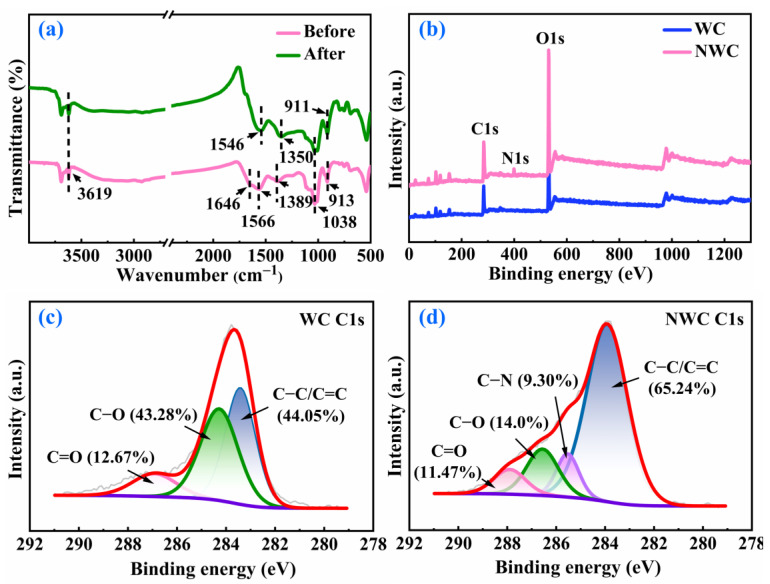
FTIR spectra (**a**); XPS survey spectra of WC and NWC (**b**), C1s of WC (**c**) and C1s of NWC (**d**).

To examine the alterations in the functional groups present on NWC, FTIR spectroscopy was conducted. As shown in Figure 2a, the spectra displayed complicated vibration bands, mainly including those of O−H, C=N, N−H, C−N, C=O, and C−O. The band at 3619 cm^−1^ corresponded to the O−H stretching vibration of the hydroxyl group [20]. The weak absorption band at 1646 cm^−1^ matched to C=N stretching. The band centered at 1566 cm^−1^ can be assigned to C=O stretching vibrations along with N−H bending [21,22]. C−O stretching was observed at approximately 1389 cm^−1^ [22]. The band at 1038 cm^−1^ was due to C−O and C−N stretching, and the sharp band at 913 cm^−1^ originated from the C=O stretching vibration [23,24]. The C=N band at 1646 cm^−1^ only appeared in NWC. The peaks at 1566 and 1038 cm^−1^ were stronger in NWC than in WC. This indicates that nitrogen was successfully doped into WC, which may improve the Pb(II) adsorption of WC [25].

XPS was employed to further investigate variations in the functional groups of NWC. The functional group content of NWC is shown in Figure 2b–d. The XPS full-scan spectrum showed that the WC surface was mainly composed of C and O. After N doping, a characteristic absorption peak of N appeared at 399.29 eV, indicating that N was added to the surface of NWC. The C1s spectra of WC could be fitted with three peaks linked to C−C/C=C, C−O, and C=O [18], while the spectra of NWC could be fitted with four peaks associated with C−C/C=C, C−N, C−O, and C=O [26]. Compared with WC, the content of C−C/C=C rose from 44.05% to 65.24% while the contents of C−O and C=O declined from 43.28% and 12.67% to 14.0% and 11.47%, respectively. In addition, the content of C−N was 9.30% in NWC. This indicated that the triethylenetetramine reacted with the oxygen-containing functional groups of WC to form C−N. Nitrogen-containing functional groups are highly electronegative and can be used as electron donors to enhance the adsorption capacity of NWC [27].

### 2.2. Adsorption of Pb(II) on NWC

#### 2.2.1. Adsorption Properties and Kinetics

**Figure 3:**
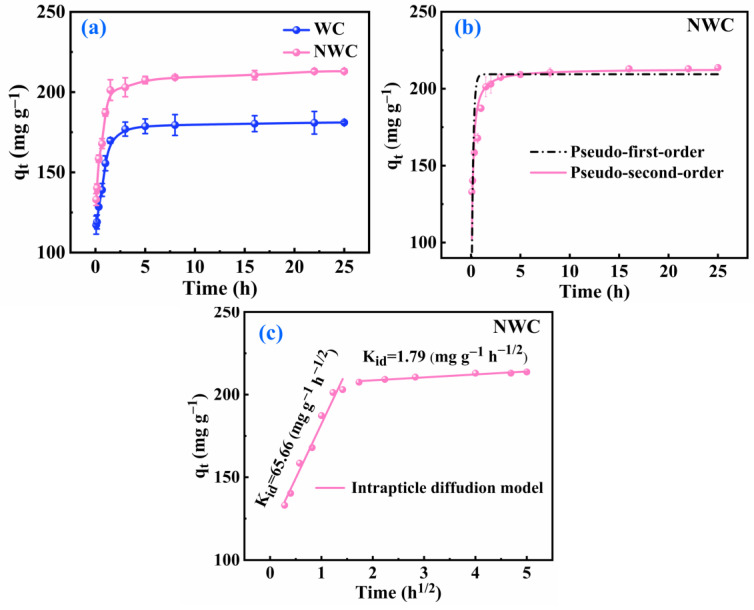
Adsorption capacity of NWC for various adsorption times (**a**); pseudo-first order and pseudo-second-order models (**b**); intraparticle diffusion kinetic plots (**c**) for Pb(II) adsorption on NWC.

An equilibrium adsorption experiment of NWC was carried out in a 200 mg L^−1^ Pb(II) solution to analyze the dynamic changes in the adsorption properties during the adsorption process. As shown in Figure 3a, rapid adsorption and slow equilibrium occurred during the Pb(II) adsorption process. The adsorption rate was rapid within the initial 1.5 h, and adsorption equilibrium was achieved within 24 h. In the rapid stage, approximately 95% of all lead was absorbed on NWC, which was ascribed to the numerous adsorption active sites provided by the oxygen and nitrogen functional groups and the significant liquid–solid concentration gradient. As Pb(II) continued to be adsorbed, the active sites became occupied, the concentration gradient decreased, the driving force weakened, and adsorption slowed as the system reached equilibrium. The adsorption capacity of Pb(II) on NWC was higher than that on WC at different times owing to the introduction of nitrogen functional groups, which enhanced complexation [28].

Kinetic fitting of the experimental data for NWC was performed employing three kinetic models, namely the pseudo-first-order, pseudo-second-order, and intraparticle diffusion types, to study the adsorption behavior of Pb(II) (Figure 3b,c). The kinetic constants calculated from the fitting results are listed in Table 1 and Table 2. As summarized in Table 1, the correlation coefficient R^2^ was close to 1 for the pseudo-second-order kinetic model, which is better than the fit of the pseudo-first-order kinetic model. This implies that the pseudo-second-order kinetic model is more suitable for describing the adsorption of Pb(II) onto both WC and NWC. This suggests that Pb(II) strongly interacts with WC and NWC and that chemisorption is a significant step in the adsorption process. Moreover, NWC had a higher theoretical equilibrium adsorption capacity than WC, which was ascribed to the contribution of the nitrogen functional groups. The presence of nitrogen in NWC increased the quantity of active adsorption sites and facilitated effective electron transfer and sharing between Pb(II) and NWC, thereby promoting the complexation capacity of Pb(II) on NWC [29].

The adsorption of Pb(II) on NWC primarily involved three processes [30]: (1) the diffusion of Pb(II) from the liquid phase to the interface of NWC-Pb(II); (2) the diffusion of Pb(II) from the interface of NWC-Pb(II) to the surface of NWC (surface diffusion); and (3) the diffusion of Pb(II) from the surface of NWC to the pores within NWC (intraparticle diffusion). Because of the vigorous shaking of Pb(II) and NWC within the shaker, the diffusion of Pb(II) from the liquid phase to the NWC-Pb(II) interface was assumed to be very rapid. Therefore, process (1) was unlikely to be the governing step for Pb(II) adsorption on NWC. A kinetic model of intraparticle diffusion was employed to further explore the diffusion mechanism of Pb. A nonlinear relationship was observed between $q_{t}$ and *t*^1/2^ (Figure 3c), indicating that intraparticle diffusion was a key factor in the adsorption of Pb(II) on NWC but was not the governing step. It was evident that the adsorption process occurred in two distinct stages. The initial stage within the first 1.5 h had a fast rate constant of 65.655 mg g^−1^ h^−1/2^, and approximately 95% of Pb(II) was adsorbed onto NWC. This primarily resulted from the surface diffusion of Pb(II) on NWC. The second adsorption stage occurred after 1.5 h, during which the adsorption equilibrium was attained and approximately 5% of Pb(II) was adsorbed on NWC. This stage primarily involves intraparticle diffusion. The diffusion rate constant of 1.787 mg g^−1^ h^−1/2^ during this process was significantly lower than that of surface diffusion. Consequently, the adsorption of Pb(II) on NWC is a composite process of surface diffusion and intraparticle diffusion, with surface diffusion being the predominant process [31].

#### 2.2.2. Adsorption Isotherms and Thermodynamics

**Figure 4:**
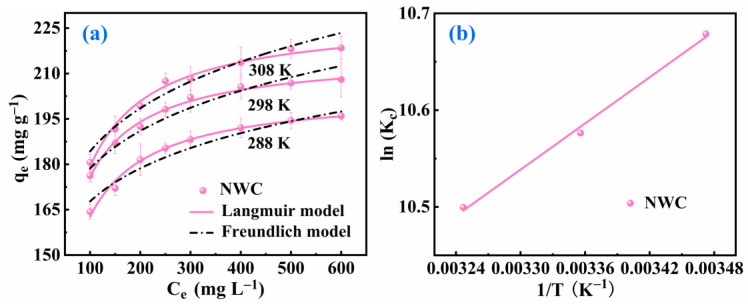
Langmuir and Freundlich models at various temperatures (**a**) and plot of lnKc versus 1/T (**b**) for Pb(II) adsorption on NWC.

The original concentration of the solution and temperature were further analyzed to determine the adsorption behavior of Pb(II) on NWC. The fitting of the experimental data at 288, 298, and 308 K was performed in accordance with the Langmuir and Freundlich isotherm models. The model constants obtained for each isotherm are presented in Table 3. As depicted in Figure 4a, the Pb (II) adsorption capacity grew with increasing solution concentration and temperature. This was because the high concentration and temperature improved the contact between Pb(II) and NWC, accelerating the adsorption process. The correlation coefficients (R^2^) were higher from the fitting of the Langmuir isotherm model at different temperatures (Table 3), indicating better fitting results compared to the Freundlich isotherm model. This indicates that the Langmuir isotherm model was more appropriate for describing the adsorption of Pb(II) on NWC and that Pb(II) adsorption on NWC formed a uniform monolayer coverage. The maximum monolayer adsorption capacities (qm) for NWC were 204.19 (288 K), 216.32 (298 K), and 228.50 (308 K) mg g^−1^, respectively, demonstrating that NWC had an efficient capacity for Pb(II) adsorption (Table S2). The increased values of qm for NWC with increasing temperature indicated an endothermic adsorption process for Pb(II) on NWC. The equilibrium parameter RL values calculated using KL at different temperatures and concentrations ranged from 0 to 1. This reveals that the adsorption of Pb(II) by NWC was advantageous under the tested conditions.

The thermodynamic properties of the reaction system were then evaluated, as shown in Figure 4b and Table 4. It was obvious that the ΔG values were negative, indicating the thermodynamical spontaneity within the adsorption process. With increasing temperature, the increasingly negative ΔG values suggested an increased driving force for Pb(II) adsorption on the NWC. ΔH had a positive value, verifying the endothermic nature within the adsorption process, and the increase in temperature promoted the adsorption process. The plus ΔS value reflected the growth of disorderliness at the solid–liquid interface throughout the process of adsorption, likely owing to the release of water molecules from the NWC surface as Pb(II) was adsorbed [32].

### 2.3. Adsorption Mechanism

According to the results of the physicochemical structure of NWC and the analysis of the adsorption kinetics, isotherms, and thermodynamics, the mechanisms of Pb(II) adsorption on NWC involve physisorption and chemisorption, and chemisorption is a significant mechanism in the adsorption process. Therefore, the change of NWC surface chemistry after Pb(II) adsorption and the contributions of different mechanisms were analyzed to further clarify the adsorption mechanism.

#### 2.3.1. Change of NWC Surface Chemistry After Pb(II) Adsorption

**Figure 5:**
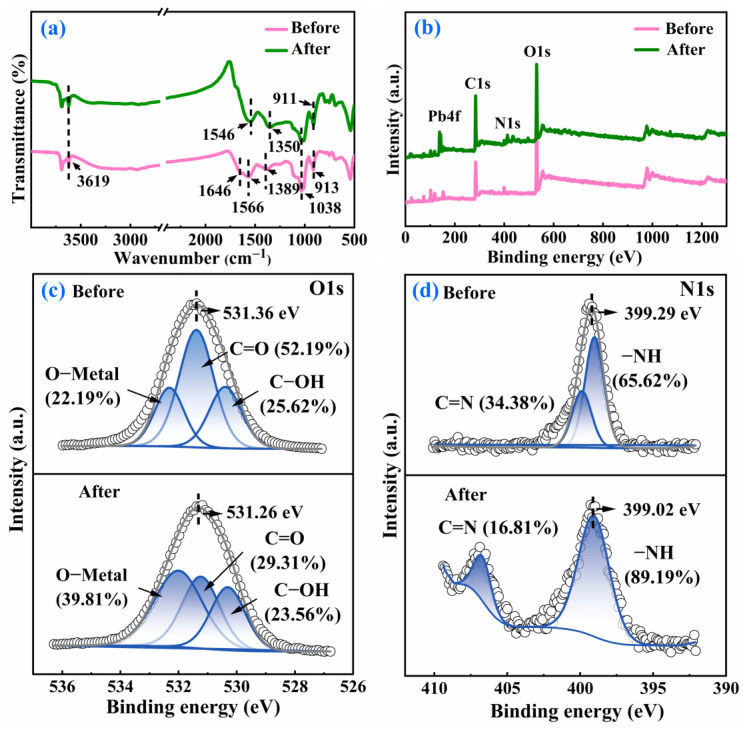
FTIR spectra (**a**); XPS survey (**b**), O1s (**c**), and N1s (**d**) spectra of NWC before and after Pb(II) adsorption.

Changes in the surface functional groups of NWC were examined using FTIR (Figure 5a). After Pb(II) adsorption, some of the characteristic peaks of NWC disappeared, shifted, or weakened, indicating interactions between NWC and Pb(II). Post-adsorption, the 1646 cm^−1^ peak originating from C=N stretching disappeared. The peak corresponding to N−H bending at 1566 cm^−1^ weakened and shifted to 1546 cm^−1^. Meanwhile, the peak at 1038 cm^−1^ matching with C−N stretching weakened. These results indicate the contribution of nitrogen-containing functional groups to Pb(II) adsorption on NWC. The C−O stretching at approximately 1389 cm^−1^ weakened and moved to 1350 cm^−1^, and the C=O stretching vibration at 913 cm^−1^ shifted to 911 cm^−1^. In addition, the intensity of the O−H stretching vibration at 3619 cm^−1^ weakened, which suggested that the oxygen-containing functional groups interacted with Pb(II), and the hydroxyl group performed an important function in the adsorption process [33].

Alterations in the elemental composition and functional groups were further determined using XPS after Pb(II) adsorption. The XPS spectra (Figure 5b) display that mainly C, O, and N elements were present on the surface of NWC pre-adsorption, whereas Pb was present on the surface of NWC after adsorption, indicating that Pb could be effectively captured by NWC from aqueous solutions. The O1s spectrum of NWC is shown in Figure 5c. The O1s binding energy underwent a shift from 531.36 eV to 531.26 eV after adsorption, suggesting that the oxygen-containing functional groups participated in the adsorption process. The spectra of O1s was fitted with three peaks attributed to C−OH, C=O, and O−Metal. The contents of C−OH and C=O reduced from 25.62% and 52.19% to 23.56% and 29.31%, respectively, while the content of O-Metal increased from 22.19% to 39.81% after Pb(II) adsorption. This can be ascribed to the complex reaction of functional groups containing oxygen, like hydroxyl and carboxyl groups, with Pb [34]. Moreover, the N1s spectrum of NWC (Figure 5d) showed that the binding energy of N1s underwent a shift from 399.29 to 399.02 eV after adsorption, implying that nitrogen-containing functional groups were engaged in the adsorption process of lead. The N1s peak was mainly deconvoluted into two peaks of −NH and C=N [35]. After absorbing Pb(II), a remarkable shift of −NH and C=N was observed along with a change in their contents. This is due to the alteration of the electron cloud density induced by the reaction of N atoms with lead [29]. The results revealed that the oxygen and nitrogen functional groups complexed with lead ions to achieve adsorption [36].

#### 2.3.2. Contributions of Different Mechanisms

**Figure 6:**
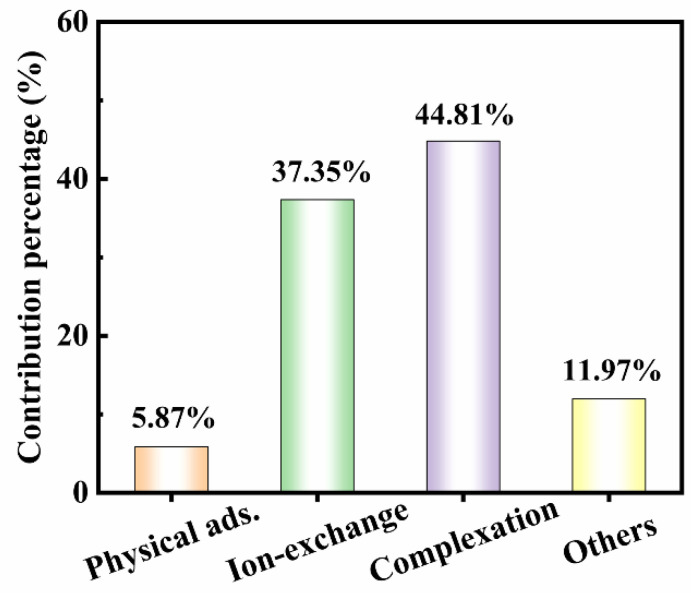
Contributions of different adsorption mechanisms.

Desorption experiments were conducted to ascertain the proportional importance of the various mechanisms in the adsorption process (Figure 6). The contribution of physical adsorption eluted by ultrapure water was only 5.87%, which was the lowest among all experiments. This indicated that there was a strong interaction between lead and NWC and it was not easy to wash off lead using ultrapure water, which further revealed that chemisorption performed an important function in this process. The percentage of lead eluted using EDTA-Na_2_ was the highest, followed by NH_4_NO_3_ desorption. This implies that complexation was the main mechanism of Pb(II) adsorption on NWC, followed by ion exchange. This complexation is owing to the coordination of oxygen and nitrogen functional groups with Pb(II). Functional groups, like hydroxyl, carboxyl, and amine groups, can function as proton donors, which are deprotonated and complexed with Pb(II). This was also supported by the XPS analysis (Figure 5). Ion exchange can be ascribed to the replacement of metal ions such as K^+^, Ca^2+^, Na^+^, and Mg^2+^ with Pb(II) [37]. Other mechanisms, primarily precipitation, were minor [38].

**Figure 7:**
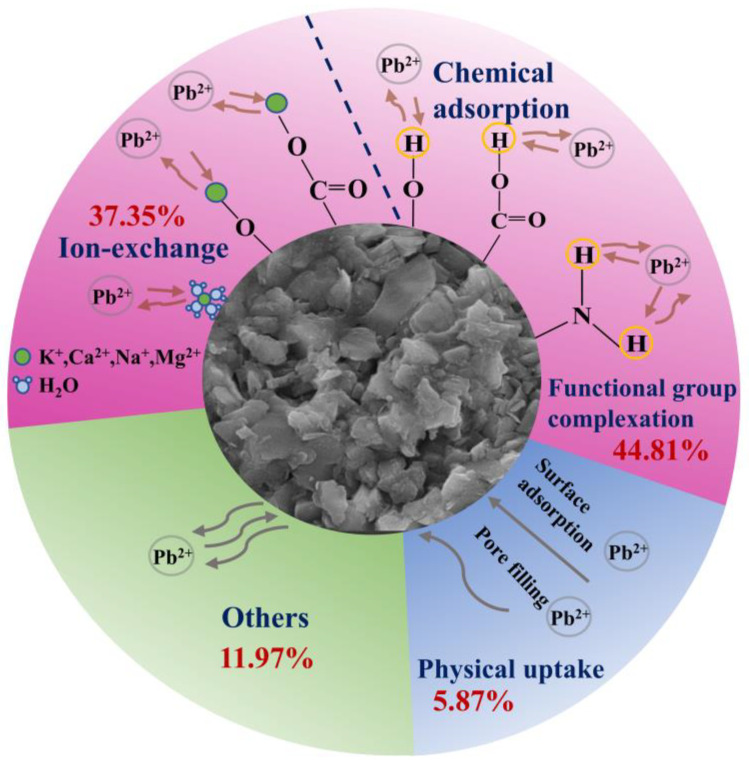
Pb(II) adsorption mechanism of NWC.

Based on the aforementioned results, the adsorption of Pb(II) on NWC involved both physical and chemical adsorption, with chemical adsorption being dominant. The adsorption process was driven by the collective action of physical uptake, emphasizing surface adsorption and pore filling, and chemisorption dominated by functional group complexation, ion exchange, and other mechanisms (Figure 7).

## 3. Material and Methods

### 3.1. Materials

The weathered coal (WC) employed in this experiment was collected from the Yongxing Coal Mine in Shanxi, ground, and sieved (<200 mesh) as the raw material. The main elemental composition of the weathered coal was shown in Table S3. It contained 9.94% Ca and a certain amount of Mg, Na, and K. The heavy metal contents of the weathered coal (Pb, Cr, Hg, Cd, Cu, Ni, Zn) were lower than the corresponding risk screening values, and the humic acid content was greater than 30%. Simulated lead wastewater was prepared with lead nitrate (analytical grade), and stock liquid of 1000 mg/L was prepared and used for subsequent adsorption experiments.

### 3.2. Preparation of N-Doped WC

The WC was added to 40 mL of ultrapure water. After modifying the pH of the system to 5 by means of 0.1 M HCl and 0.1 M NaOH solutions, 2.6 mL triethylenetetramine was added. The mixture was sonicated at 350 W for 65 min. The solution was then filtered, and the produced NWC was rinsed with ultrapure water until neutral and dried.

### 3.3. Characterization

The surface morphologies of the samples were observed by means of scanning electron microscopy (SEM; MIRA4, Tescan, Brno, Czech Republic). The specific surface areas and pore structures of the samples were determined by means of an ASAP 2460 physical adsorption analyzer (Micromeritics, Norcross, GA, USA). The samples were outgassed at 90 °C for 1 h and then 120 °C for 12 h before N_2_ physisorption measurements. The functional groups of the readied samples were investigated using a Nicolet iS20 Fourier transform infrared (FTIR) spectrometer (Thermo Fisher Scientific, Waltham, MA, USA), which was recorded on the samples using the potassium bromide/sample pellet technique at ambient temperature at a ratio of 300 to 1. The number of scans and resolution were 32 and 4 cm^−1^. The elemental compositions and chemical states of the samples were detected using X-ray photoelectron spectrometry (XPS) from (K-alpha, Thermo Fisher Scientific, Waltham, MA, USA). The charge correction of the samples was performed using the binding energy of C1s at 284.80 eV as the energy standard. Peak deconvolution was analyzed by the XPS peak 4.1 software [39].

### 3.4. Performance Evaluation

Batch adsorption experiments were carried out to assess the adsorption effect of Pb(II) using NWC. Firstly, 0.02 g of NWC was put into 50 mL of wastewater at an original concentration of 200 mg L^−1^. The mixes were sealed and shaken under a constant temperature (298 K) at 180 rpm min^−1^ for different time periods. After the completion of adsorption, the supernatant was passed through a microfilter with a 0.45 μm pore size, and the Pb(II) concentrations in the filtrates were then determined by means of the atomic absorption spectroscopy (AAS; 240/240FS, Agilent, Santa Clara, CA, USA). In addition, isotherm experiments were performed using 50 mL of various concentrations of Pb(II) solution with 0.02 g of NWC, and the mixtures were shaken at different constant temperatures at 180 rpm for 25 h. The original pH of the solution was 4, and a blank test was conducted without any absorbent. All experiments were conducted in triplicate. The quantity of Pb(II) adsorbed by the NWC was determined using Equation (1).

(1) $$q_{e}=(C_{0}-C_{e})V/W$$

where *q_e_* is the adsorption quantity at equilibrium (mg g^−1^), *C*_0_ is the original concentration of the solution (mg L^−1^), *C_e_* is the concentration of the equilibrated liquid-phase after the adsorption (mg L^−1^), *V* is the volume of the solution (L), and *W* is the mass of the adsorbent (g). Adsorption kinetics, isotherms, and thermodynamic analyses were conducted (for more information, see Text S1–S3) to analyze the adsorption properties and clarify the adsorption mechanism.

### 3.5. Desorption Experiments

The adsorption mechanism of Pb(II) on NWC was quantitatively assessed using the following equation [40,41]: NWC (0.02 g) was put into 50 mL of a Pb(II) solution at an original concentration of 200 mg L^−1^. After adsorption was complete, the solid was cleaned with a small amount of ultrapure water, sequentially transferred to three eluent solutions (ultrapure water (pH = 4), NH_4_NO_3_ and EDTA-Na_2_) and shaken to achieve equilibrium. The three supernatants were filtered and analyzed. The contribution of each adsorption mechanism was determined from the desorption percentage (*D*%) calculated by means of Equation (2). All experiments were conducted in triplicate.

(2) $${D=C}_{E}/\left(C_{0}-C_{e}\right)\;\times \;100$$

where *C_E_* is the concentration of the equilibrated liquid phase in the elution solution (mg L^−1^). Desorption using water is known as physical adsorption *(D_p_*). Desorption by NH_4_NO_3_ follows an ion-exchange mechanism (*D_I_*). Desorption by EDTA-Na_2_ was attributed to the complexation mechanism (*D_c_*). The other mechanism (*D_O_*) was calculated by subtracting *D_p_*, *D_I_*, and *D_c_* from 100%.

## 4. Conclusions

NWC was prepared using WC as the raw material and triethylenetetramine as the N-doping agent to achieve the high-value utilization of WC and improve its Pb(II) adsorption performance. The physicochemical structure of NWC exhibited successful N-doping via the introduction of functional groups containing nitrogen with high electronegativity, which increased the adsorption capacity of Pb(II) by 18% compared to WC. The maximum monolayer adsorption capacity of NWC was 216.32 mg g^−1^ (25 °C), making it a promising adsorbent for the elimination of Pb(II). The adsorption process was determined to be spontaneous and endothermic, and chemisorption is an important step in this process. The adsorption mechanism of NWC involves physical adsorption, ion exchange, complexation, and others. Complexation was the predominant adsorption mechanism, accounting for 44.81% of the adsorption process, and it was attributed to the coordination of the hydroxyl, carboxyl, and amine groups with Pb(II). Therefore, NWC exhibits great potential for wastewater treatment.

## Figures and Tables

**Table 1:** Kinetic parameters for Pb(II) adsorption on WC and NWC.

Samples	Pseudo-First Order Model			Pseudo-Second Order Model		
	k_1_ (h^−1^)	q_e_ (mg g^−1^)	R^2^	k_2_ (g mg^−1^ h^−1^)	q_e_ (mg g^−1^)	R^2^
WC	3.879	178.19	0.936	7.350	181.83	0.977
NWC	6.283	209.39	0.684	11.480	212.91	0.920

**Table 2:** Intraparticle diffusion kinetic parameters for Pb(II) adsorption on NWC.

Samples		Weber–Morris Intraparticle Diffusion Model		
		k_id_ (mg g^−1^ h^−1/2^)	C	R^2^
NWC	I	65.655	116.590	0.975
	II	1.787	205.051	0.949

**Table 3:** Langmuir and Freundlich constants for Pb(II) adsorption on NWC.

Sample	T	Langmuir Model			Freundlich Model		
	(K)	q_m_ (mg g^−1^)	K_L_ (L mg^−1^)	R_1_^2^	K_F_ (mg g^−1^ (L mg^−1^)^1/n^)	1/n	R_2_^2^
NWC	288	204.19	0.04342	0.991	110.256	0.091	0.952
	298	216.32	0.0392	0.996	114.275	0.097	0.960
	308	228.50	0.03663	0.984	112.278	0.107	0.965

**Table 4:** Thermodynamic parameters for Pb(II) adsorption on NWC at various temperatures.

Sample	T (K)	∆G (KJ mol^−1^)	∆H (KJ mol^−1^)	∆S (KJ mol^−1^ K^−1^)
NWC	288	−25.569	6.920	0.066
	298	−26.203		
	308	−26.886		

## Data Availability

Data are contained within the article.

## Supplementary Materials

The following supporting information can be downloaded at: https://www.mdpi.com/article/10.3390/molecules29235589/s1, Text S1: Adsorption kinetic; Text S2: Adsorption isotherm; Text S3: Adsorption thermodynamic; Table S1: Pore structural parameters of WC and NWC; Table S2: The comparison of the adsorption capacity for Pb^2+^ on NWC with other adsorbents reported in literature; Table S3: The main elemental composition of WC (%). References [2,4,5,8,29,31,42,43,44,45,46,47,48] are cited in the Supplementary Materials.
