# Supplementary material for: Nitrogen-Doped Weathered Coal for the Efficient Adsorption of Lead: Adsorption Performance and Mechanisms
Source: Molecules. 2024 Nov 26;29(23):5589. doi: 10.3390/molecules29235589 (PMC11643971; doi:10.3390/molecules29235589)
Supplement: Supplementary file 1 [file molecules-29-05589-s001.zip › molecules-3322645-supplementary.pdf]

Supplementary Materials

Nitrogen-doped Weathered Coal for Efficient Adsorption of Lead: Adsorption Performance and Mechanisms

Xiaojing Chen [1,2,3,*], Xiaobing Jin [1], Chi Zhang [1], Zile Jiao [4], Zhiping Yang [1,2], Ke Wang [1,2], Jianhua Li [1,2] and Qiang Zhang [1,2,*]

1 Shanxi Agricultural University, Taiyuan 030031, China

2 Shanxi Province Key Laboratory of Soil Environment and Nutrient Resources, Taiyuan 030031, China

3 Institute of Loess Plateau, Shanxi University, Taiyuan 030006, China

4 Beijing Forestry University, Beijing 100083, China

* Correspondence: chen-xiao-jing1985@sxau.edu.cn (X.J. Chen), zhangqiang0351@163.com (Qiang Zhang)

Supporting Texts

Text S1. Adsorption kinetic

The adsorption kinetics of Pb(II) by NWC were studied with pseudo-first-order kinetic model, pseudo-second-order kinetic model, and intraparticle diffusion kinetic model, which were given by Equation S(1), S(2) and S(3). These models were used to determine the adsorption rate and the mechanism controlling the adsorption process [1, 2].

$$q_t=q_e(1-exp(-k_1t)) \quad S(1)$$

where q_e (mg g^{-1}) and q_t (mg g^{-1}) respectively represent the adsorption quantity at the equilibrium and the time at t, and k_1 (h^{-1}) stands for the pseudo-first-order rate constant. The values of k_1 and q_e can be obtained from the curve fitting.

$$t/q_t=1/k_2q_e^2+t/q_e=1/V_0+t/q_e \quad S(2)$$

where V_0 (mg g^{-1} h^{-1}) represents the initial adsorption rate, and k_2 (g mg^{-1} h^{-1}) denotes the pseudo-second-order rate constant.

$$q_t=k_3t^{1/2}+C \quad S(3)$$

where k_3 (mg g^{-1} h$^{-1/2}$) represents the rate constant of intraparticle diffusion, and C is a constant denoting the thickness of boundary layer (mg g^{-1}). The values of k_3 and C are calculated by the slope and the intercept from the plot of q_t against $t^{1/2}$. When the intraparticle diffusion dictates the kinetics of adsorption process, the plot of q_t against $t^{1/2}$ becomes a straight line which passes through the original point, and the slope represents the rate constant of intraparticle diffusion.

Text S2. Adsorption isotherm

2.1 Langmuir isotherm model

$$C_e/q_e=1/q_m k_L+C_e/q_m \quad S(4)$$

$$R_L=1/(1+k_L C_0) \quad S(5)$$

Where q_e (mg g^{-1}) represents the adsorption quantity of Pb(II) per unit mass of adsorbent at equilibrium, C_e (mg L^{-1}) is the Pb(II) concentration at equilibrium, and k_L stands for the Langmuir adsorption constant (L mg^{-1}), q_m represents the theoretical maximum adsorption capacity (mg g^{-1}), and C_0 is the initial concentration of Pb(II) (mg L^{-1}) [3].

2.2 Freundlich isotherm model

$$q_e=k_F C_e^{1/n} \quad S(6)$$

Where k_F and n are the adsorption constants (mg g^{-1} (L mg^{-1})$^{1/n}$) in Freundlich model, correlated with the adsorption capacity and intensity [4].

Text S3. Adsorption thermodynamic

$$\Delta G=-RT\ln K_c \quad S(7)$$

$$\ln K_c=-\Delta H/RT+\Delta S/R \quad S(8)$$

Where R is the gas constant (8.314 J mol^{-1} K^{-1}), and T (K) is the thermodynamic absolute temperature. The Langmuir adsorption constant, K_c, can be found from the plot of C_e/q_e versus C_e (converted to dimensionless constants using standard concentrations) [5].

Table S1 Pore structural parameters of WC and NWC

Samples	S_{BET} (m^2 g^{-1})	S_{mic} (m^2 g^{-1})	S_{mes} (m^2 g^{-1})	V_{mic} (cm^3 g^{-1})	V_t (cm^3 g^{-1})
WC	4.07	0.82	3.34	0.0004	0.009
NWC	1.73	1.89	1.23	0.0007	0.004

Table S2 The comparison of the adsorption capacity for Pb^{2+} on NWC with other adsorbents reported in literature

Adsorbent	T (°C)	Q_m (mg g^{-1})	Reference
Bentonit-Chitosan composites	21	94.60	[6]
Araucaria gum/calcium alginate composite beads	25	149.95	[7]
La-MOF decorated PANI	25	185.19	[8]
Sulfate-functionalized Fe-based MOF	25	504	[9]
Electrochemical method to activate coffee ground biochar	25	67.07	[10]
Alginate/gum composite	25	296.01	[11]
Nitrogen-doped biochar	25	130.87	[12]
Straw biochar-loaded N-doped carbon aerogel	25	205.07	[13]
N-doped weathered coal	25	216.32	This study

Table S3 The main elemental composition of WC (%)

Si	Al	Ca	Sx	Fe	Mg	Ti	Na	K	Px	Sr	Zr
19.34	18.39	9.94	1.24	1.56	0.58	0.55	0.46	0.35	0.13	0.13	0.04

References

1. Meng, K.K.; Wang, Y.F.; Liu, F.F.; Zhan, Q.P.; Zhao, L.Y. Effect of modifications on structure, physicochemical properties and lead ions adsorption behavior of dietary fiber of Flammulina velutipes. Food Chem. 2025, 464, 141597. https://doi.org/10.1016/j.foodchem.2024.141597.
2. Zhao, F.; Su, C.H.; Yang, W.X.; Han, Y.; Luo, X.L.; Li, C.H.; Tang, W.Z.; Yue, T.L.; Li, Z.H. In-situ growth of UiO-66-NH_2 onto polyacrylamide-grafted nonwoven fabric for highly efficient Pb(II) removal. Appl. Surf. Sci. 2020, 527, 146862. https://doi.org/10.1016/j.apsusc.2020.146862.
3. Wang, G.G.; Chen, C.Y.; Li, J.Q.; Lan, Y.P.; Lin, X.; Chen, J.H. Conversion of phosphogypsum into porous calcium silicate hydrate for the removal and recycling of Pb(II) and Cd(II) from wastewater. Molecules 2024, 29, 2665. 10.3390/molecules29112665..
4. Güzel, F.; Yılmaz, C. Synthesis, characterization, and lead (II) sorption performance of a new magnetic separable composite: $MnFe_2O_4$@wild plants-derived biochar. J. Environ. Chem. Eng. 2021, 9, 104567. https://doi.org/10.1016/j.jece.2020.104567.
5. Zhou, Y.; Li, F.; He, Y.; Qiu, Y.W.; Zhou, Z.C.; Bai, H.P.; Jiang, F.Z.; Wang, S.X.; Yang, X.J. Lanthanum(III)-2,5-Pyrazinedicarboxylate framework as an antibacterial adsorbent for highly efficient and selective capture of Pb(II) and Cd(II) from wastewater. Sep. Purif. Technol. 2024, 345, 127329. https://doi.org/10.1016/j.seppur.2024.127329.

6. Majiya, H.; Clegg, F.; Sammon, C. Bentonite-Chitosan composites or beads for lead (Pb) adsorption: Design, preparation, and characterization. Appl. Clay Sci. 2023, 246, 107180. https://doi.org/10.1016/j.clay.2023.107180.
7. Khoj, M.A,; Hassan, A.F.; Awwad, N.S.; Ibrahium, H.A.; Shaltout, W.A. Fabrication and characterization of Araucaria gum/calcium alginate composite beads for batch and column adsorption of lead ions. Int. J. Biol. Macromol. 2024, 255, 128234. https://doi.org/10.1016/j.ijbiomac.2023.128234
8. Govarthanan, M.; Jeon, C.H.; Kim, W. Synthesis and characterization of lanthanum-based metal organic framework decorated polyaniline for effective adsorption of lead ions from aqueous solutions. Environ. Pollut. 2022, 303, 119049. https://doi.org/10.1016/j.envpol.2022.119049.
9. Wang, R.D.; Zhang, W.Q.; Lv, H.B.; Chen, Y.T.; Wang, L.; Zhou, S.H.; Du, L.; Zhao, Q.H. Sulfate-functionalized Fe-based MOF for removal of Pb(II) and $NO3^{-}$ in industrial wastewater. J. Environ. Chem. Eng. 2024, 12, 112167. https://doi.org/10.1016/j.jece.2024.112167.
10. Kim, J.G.; Kim, H.B.; Baek, K. Novel electrochemical method to activate biochar derived from spent coffee grounds for enhanced adsorption of lead (Pb). Sci. Total Environ. 2023, 886, 163891. https://doi.org/10.1016/j.scitotenv.2023.163891.
11. Elwakeel, K.Z.; Ahmed, M.M.; Akhdhar, A.; Alghamdi, H.M.; Sulaiman, M.G.M.; Hamza, M.F.; Khan, Z.Y. Effect of the magnetic core in alginate/gum composite on adsorption of divalent copper, cadmium, and lead ions in the aqueous system. Int. J. Biol. Macromol. 2023, 253, 126884; https://doi.org/10.1016/j.ijbiomac.2023.126884.

12. Jiang, S.Y.; Yan, L.L.; Wang, R.K.; Li, G.H.; Rao, P.H.; Ju, M.C.; Jian, L.; Guo, X.; Che, L. Recyclable nitrogen-doped biochar via low-temperature pyrolysis for enhanced lead(II) removal. Chemosphere 2022, 286, 131666. https://doi.org/10.1016/j.chemosphere.2021.131666.
13. Li, G.H.; Xia, C.G.; Cheng, R.; Lan, J.R.; Chen, F.Y.; Li, X.L.; Li, S.Y.; Chen, G.A.; Zeng, T.Y.; Hou, H.B. Passivation of multiple heavy metals in lead–zinc tailings facilitated by straw biochar-loaded N-doped carbon aerogel nanoparticles: Mechanisms and microbial community evolution. Sci. Total Environ. 2022, 803, 149866. https://doi.org/10.1016/j.scitotenv.2021.149866.
